# A barium complex of a phenoxazinone sulfonate dye

**DOI:** 10.1107/S2056989025008242

**Published:** 2025-09-23

**Authors:** Jared T. Doney, Philip J. Squattrito, Christopher G. Gianopoulos

**Affiliations:** a Department of Chemistry and Biochemistry Central Michigan University,Mount Pleasant Michigan 48859 USA; b Department of Chemistry and Biochemistry, University of Toledo, Toledo, OH 43606, USA; Texas A & M University, USA

**Keywords:** crystal structure, metal salt, phenoxazinone sulfonate derivative, hydrogen bonding

## Abstract

An aqueous reaction of barium hydroxide and 3-amino-4-hy­droxy­benzene­sulfonic acid yielded a very small amount of an unexpected product, bis­(2-amino-3-oxo-3*H*-phenoxazine-8-sulfonato)­tetra­aqua­barium, [Ba(C_12_H_7_N_2_O_5_S)_2_(H_2_O)_4_], (I). The compound crystallizes in the triclinic space group *P*1 with all atoms on general positions. The barium cation is 8-coordinate with three sulfonate O atoms, one carbonyl O atom, and four water mol­ecules at Ba—O distances of 2.716 (2) to 2.807 (1) Å. The two independent anions have essentially planar phenoxazinone systems with an angle of *ca* 2.4° between them. The extended structure has columns of nearly parallel anions that are bridged by the hydrated barium cations so that the overall motif consists of alternating layers of inorganic cations and organic anions. The packing is reinforced by O—H⋯O and N—H⋯O hydrogen bonds involving the coordinated water mol­ecules, amine groups, and sulfonate groups. This is the first reported structure of a metal salt of a phenoxazinone sulfonate derivative. The condensation of 3-amino-4-hy­droxy­benzene­sulfonic acid to form the phenoxazinone is known to occur in an enzyme-catalyzed reaction, though how or when it occurred here is uncertain.

## Abstract 2

An aqueous reaction of barium hydroxide and 3-amino-4-hy­droxy­benzene­sulfonic acid yielded a very small amount of an unexpected product, bis­(2-amino-3-oxo-3*H*-phenoxazine-8-sulfonato)­tetra­aqua­barium. The extended structure has columns of nearly parallel anions that are bridged by the hydrated barium cations so that the overall motif consists of alternating layers of inorganic cations and organic anions.

## 1. Chemical context

Organo­sulfonate anions have been an active focus as building blocks for metal–organic framework (MOF) structures for the past few decades (Zhang & Fei, 2019 ▸; Dey *et al.*, 2014 ▸; Shimizu *et al.*, 2009 ▸). As part of our continuing inter­est in metal organo­sulfonate salts (Bettinger *et al.*, 2022 ▸), we recently attempted to prepare a barium 3-amino-4-hy­droxy­benzene­sulfonate compound. No crystals of the target product were obtained but a small yield of an unexpected product, bis­(2-amino-3-oxo-3*H*-phenoxazine-8-sulfonato)­tetra­aqua­barium, (I), was discovered and structurally characterized. We subsequently learned that the starting 3-amino-4-hy­droxy­benzene­sulfonic acid (i) can be converted to the observed 2-amino-3-oxo-3*H*-phenoxazine-8-sulfonic acid (ii) *via *an enzyme produced by a fungus (Forte *et al.*, 2010 ▸).
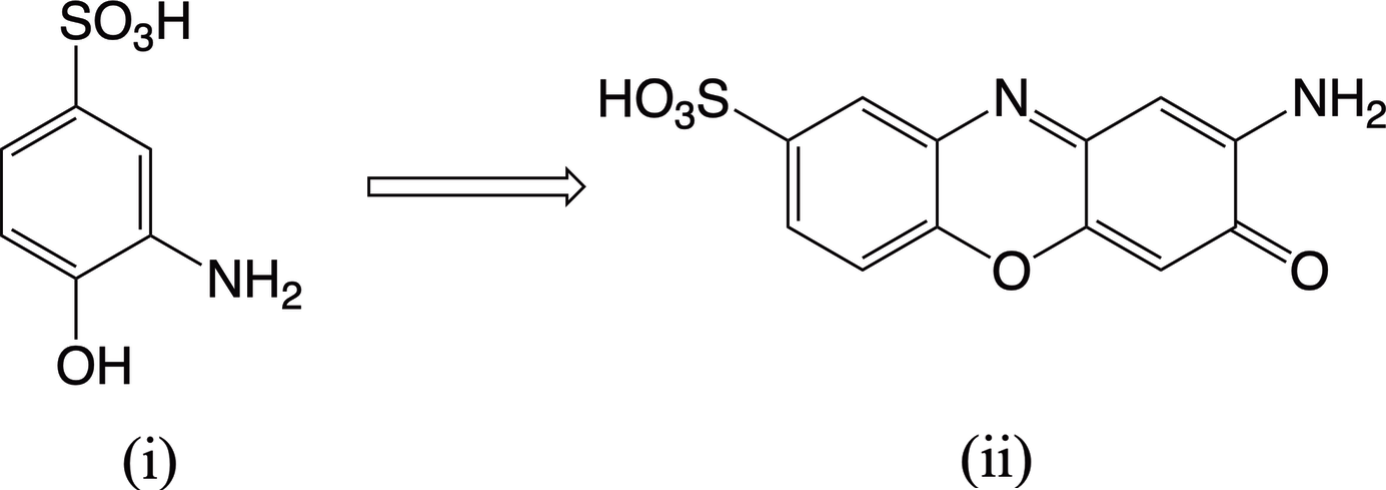


The product sulfonate belongs to a class of tricyclic organic compounds that are of inter­est as dyes (Bruyneel *et al.*, 2010 ▸) and pharmacological agents (Graf *et al.*, 2007 ▸; Zhou *et al.*, 2021 ▸). To our knowledge, this is the first crystal structure of a metal complex of a sulfonate derivative containing the phenoxazine ring system.
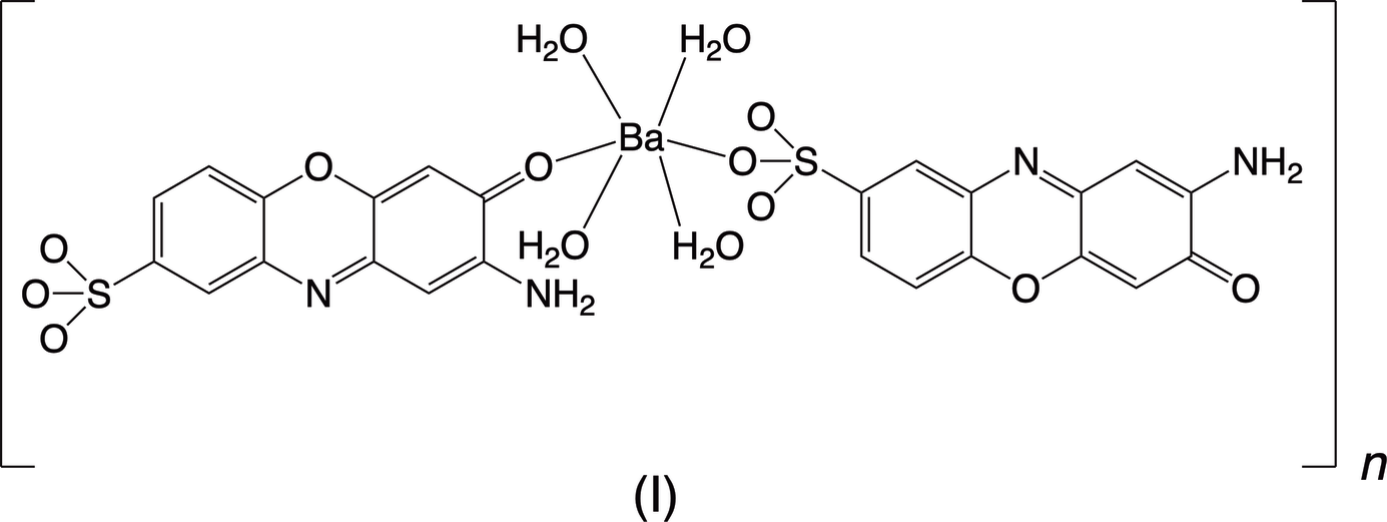


## 2. Structural commentary

**Figure 1:**
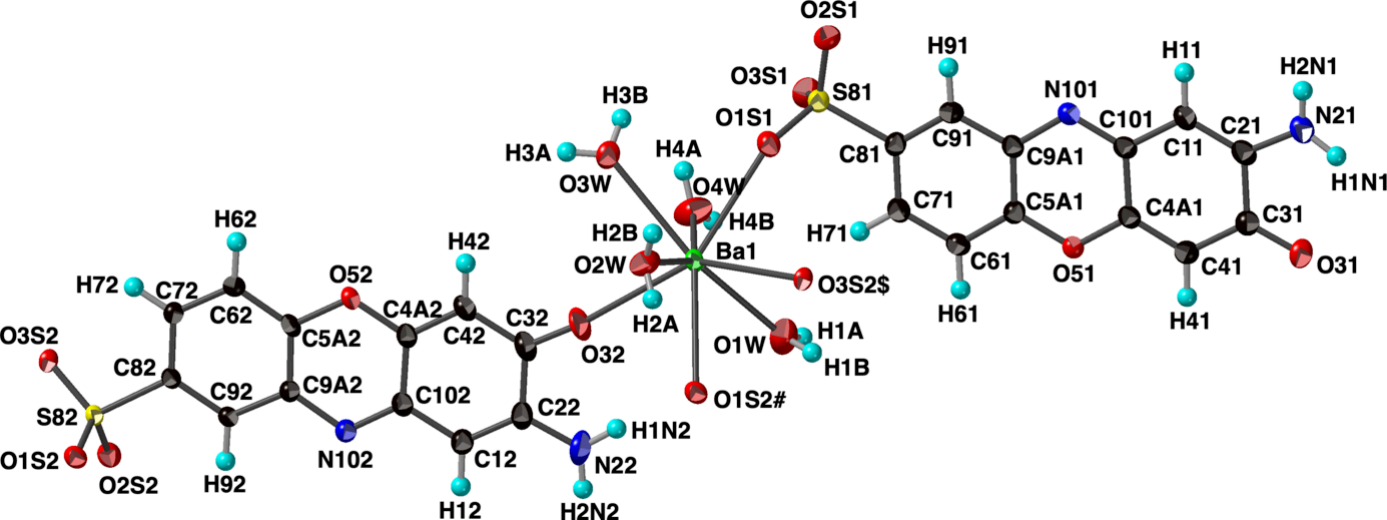
The mol­ecular structure of (I), showing the atom-numbering scheme. Displacement ellipsoids are shown at the 70% probability level and hydrogen atoms are shown as small spheres of arbitrary radii. Symmetry-equivalent oxygen atoms are included to show the complete coordination environment of the cation. [Symmetry codes: (#) −*x* + 1, −*y* + 1, −*z* + 1; ($) *x*, *y*, *z* + 1.]

**Figure 2:**
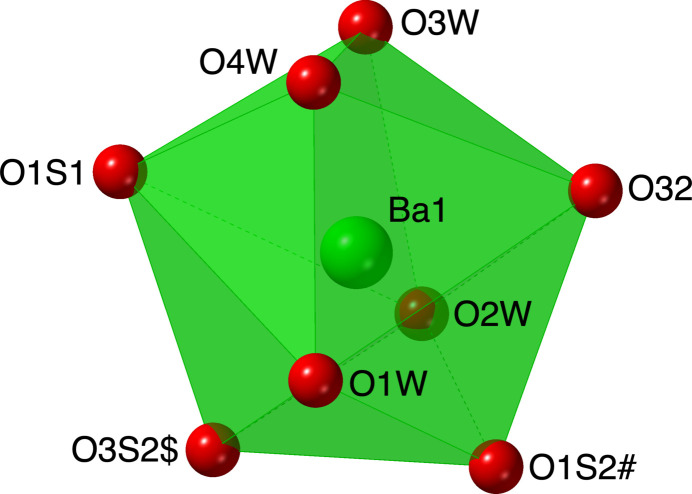
The coordination geometry of barium in (I). Barium and oxygen atoms are shown as spheres of arbitrary radii. [Symmetry codes: (#) −*x* + 1, −*y* + 1, −*z* + 1; ($) *x*, *y*, *z* + 1.]

The reaction of barium hydroxide octa­hydrate and 3-amino-4-hy­droxy­benzene­sulfonic acid monohydrate in water with gentle heating and slow evaporation to dryness at ambient temperature and pressure repeatedly produces a polycrystalline residue. In one such reaction, a couple of small clusters of red needle-shaped crystals were found. The structure determination revealed the crystals to be bis­(2-amino-3-oxo-3*H*-phenoxazine-8-sulfonato)­tetra­aqua­barium, Ba(C_12_H_7_N_2_O_5_S)_2_(H_2_O)_4_, (I). The compound crystallizes in the triclinic space group *P*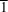 with the asymmetric unit consisting of one barium cation, two 2-amino-3-oxo-3*H*-phenoxazine-8-sulfonate anions, and four coordinated water mol­ecules, all in general positions. The barium ion has an eightfold coordination of three sulfonate O atoms from three different anions, one oxo O atom from a fourth anion, and four water mol­ecules (Fig. 1 ▸). This mix of sulfonate and water in the coordination sphere and the Ba—O distances [2.716 (2)–2.807 (1) Å] are consistent with other barium sulfonate complexes (Gunderman *et al.*, 1997 ▸; Gao *et al.*, 2005*a* ▸; Black *et al.*, 2019 ▸). The coordination geometry is somewhat irregular but could be described as either a triangular dodeca­hedron or a square anti­prism (Lippard & Russ, 1967 ▸). There are two trapezoids around the cation formed by the four water mol­ecules and the three sulfonate and one oxo O atoms, respectively (Fig. 2 ▸). The angle between these planes is *ca.* 88°. This angle for the ideal dodeca­hedron (point symmetry *D*_2*d*_) is 90°, while it is 77.4° for the ideal square anti­prism (point symmetry *D*_4*d*_). On this basis, the coordination environment in (I) is better described as dodeca­hedral. Barium shows a wide diversity of coordination environments in sulfonate systems with eight-, nine- and tenfold coordination being the most common (Wu *et al.*, 2011 ▸; Gardner *et al.*, 2020 ▸). The two independent 2-amino-3-oxo-3*H*-phenoxazine-8-sulfonate anions are both highly planar with C—C, C—O, and C—N distances that agree with those found in the related 2-amino-3-oxo-7-meth­oxy-3*H*-phenoxazine (Buckley *et al.*, 1982 ▸). They report a fold angle across the O5⋯N10 line of 5°, however the similar angles in (I) are *ca.* 1° for anion 1 and 0.5° for anion 2. The angle between the two anion planes is *ca.* 2.4°. The main conformational difference between the anions is a roughly 30° rotation of the sulfonate group [torsion angles: anion 1 C71—C81—S81—O1*S*1 = 52.6 (2)°, anion 2 C72—C82—S82—O3*S*2 = 21.3 (2)°]. Anion 1 only bonds to the Ba^2+^ cation through one sulfonate O atom (O1*S*1), while each anion 2 bonds to three different cations through O32 (shown in Fig. 1 ▸) and sulfonate O atoms O1*S*2 and O3*S*2.

## 3. Supra­molecular features

**Figure 3:**
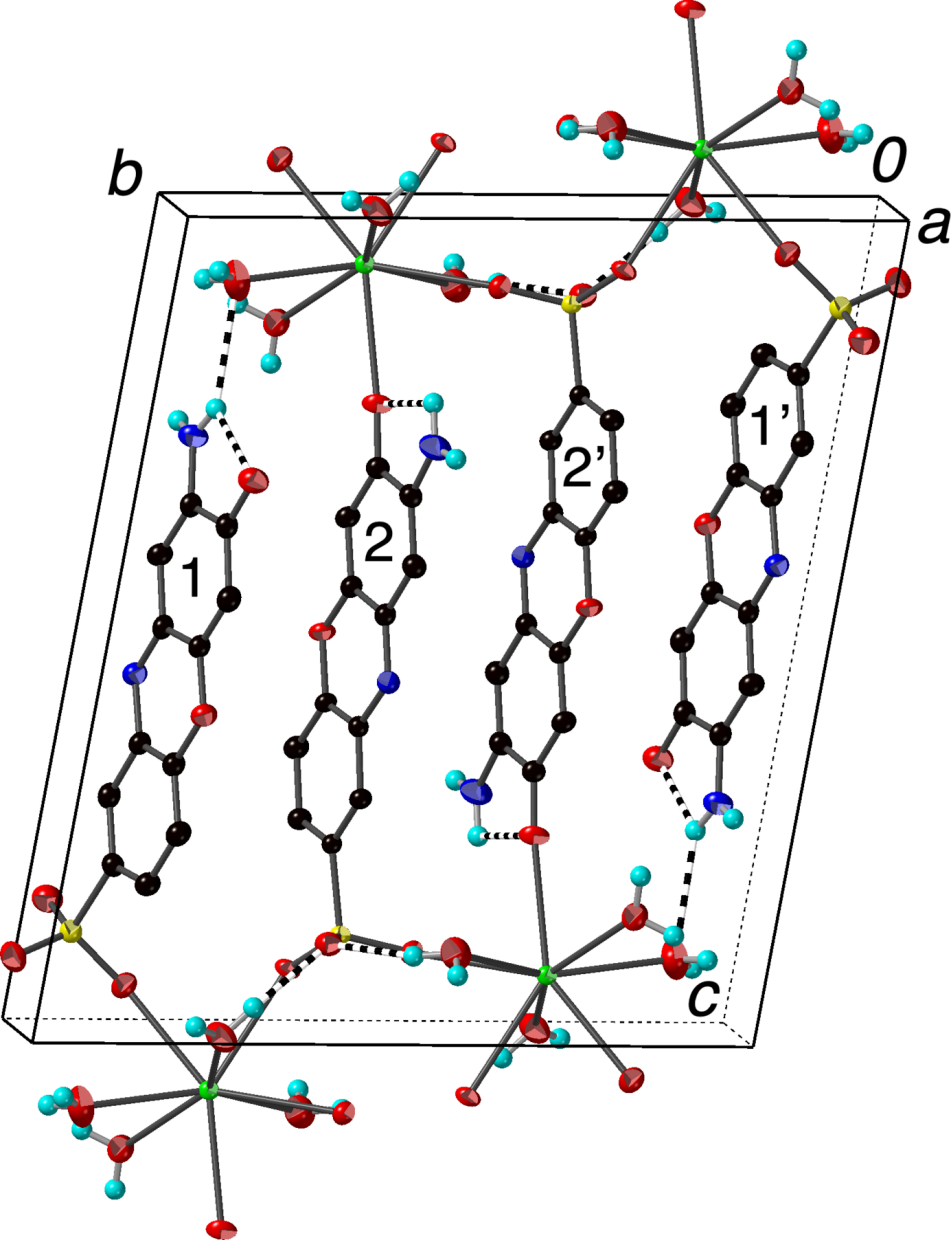
Packing diagram of (I) with the outline of the unit cell. The alternating layers of barium cations and 2-amino-3-oxo-3*H*-phenoxazine-8-sulfonate anions are evident. The independent anions are marked 1 and 2, correlating with the last digit in the atom labels. O—H⋯O and N—H⋯O hydrogen bonds are shown as striped cylinders. Anions related by the inversion at the center of the cell are marked 2′ and 1′. H atoms bonded to C atoms have been omitted. Displacement ellipsoids are drawn at the 70% probability level.

The packing in (I) features layers of nearly parallel 2-amino-3-oxo-3*H*-phenoxazine-8-sulfonate anions in the *ab* plane with the barium cations and water mol­ecules in between (Fig. 3 ▸). These layers stack along the *c-*axis direction. The two independent anions (marked 1 and 2 in Fig. 3 ▸) both have their sulfonate groups oriented down relative to the stacking direction, while the anions on the other side of the cell (2′ and 1′ in Fig. 3 ▸) are flipped in keeping with the inversion symmetry. This detail is different from most arene mono­sulfonate systems we have examined, where the sulfonate groups alternate up-down-up-down and are usually all symmetry-related (Genther *et al.*, 2007 ▸). If one looks only at the phenoxazine rings and ignores the functional groups, 1 and 2 are approximately related by inversion at the midpoint between them, while 2′ and 1′ are translationally related to 1 and 2, respectively. Thus, the approximate symmetry of the organic moieties is higher than the overall structure (Brock, 2022 ▸). This is in keeping with what is found in the neutral phenoxazine mol­ecules 2-amino-3*H*-phenoxazin-3-one (Nie & Xu, 2002 ▸) and 2-amino-7-meth­oxy-3*H*-phenoxazin-3-one (Buckley *et al.*, 1982 ▸), both of which have four symmetry-related mol­ecules in their unit cells. The approximate symmetry in (I) is broken by the positioning of the functional groups, presumably to accommodate the coordination of the barium ions and the hydrogen bonding scheme. As can be seen in Fig. 3 ▸, anion 2 bridges two cations within a layer across the inversion center at 0.5, 0.5, 0 and bridges cations in adjacent layers *via* sulfonate and oxo O atom coordination. Although anion 1 is only coordinated to a single cation, it does form an N—H⋯O hydrogen bond to a coordinated water mol­ecule in the next layer (Table 1 ▸).

**Figure 4:**
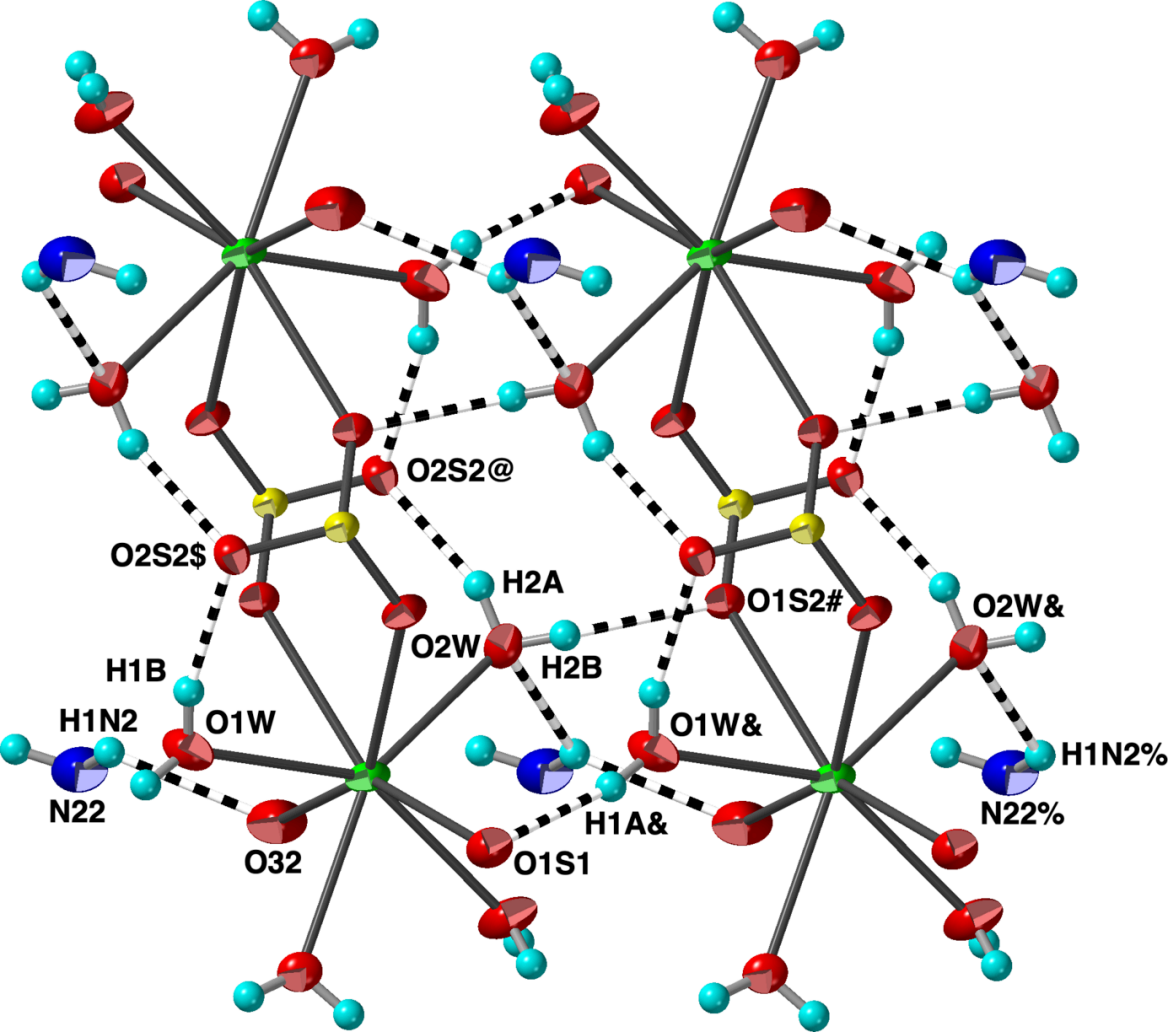
A portion of the hydrogen-bonding network with O—H⋯O and N—H ⋯O hydrogen bonds shown as striped cylinders. C atoms and their bonded H atoms have been omitted. Displacement ellipsoids are drawn at the 70% probability level. View is approximately onto the (001) plane. [Symmetry codes: ($) *x*, *y*, *z* + 1; (@) −*x* + 1, −*y* + 1, −*z* + 1; (#) −*x* + 2, −*y* + 1, −*z* + 1; (&) *x* + 1, *y*, *z*; (%) *x* + 2, *y*, *z*.]

The structure is reinforced by an extensive network of strong (H⋯O *ca.* 1.9–2.2Å) approximately linear O—H⋯O hydrogen bonds (Table 1 ▸, Fig. 4 ▸) involving the coordinated water mol­ecules and sulfonate groups. All of the water H atoms participate in such inter­actions, while five sulfonate O atoms and one oxo O atom function as hydrogen-bond acceptors. The amine groups also participate in somewhat longer N—H⋯O hydrogen bonds with water and sulfonate O atoms (Figs. 3 ▸ and 4 ▸, Table 1 ▸).

## 4. Database survey

A search of the Cambridge Structural Database (CSD, Version 5.42, update of November 2020; Groom *et al.*, 2016 ▸) for the 2-amino-3*H*-phenoxazin-3-one core yielded 17 hits. These include 2-amino-3*H*-phenoxazin-3-one itself (refcode XINYUO; Nie & Xu, 2002 ▸), 2-amino-7-meth­oxy-3*H*-phenoxazin-3-one (refcode BAXVOL; Buckley *et al.*, 1982 ▸) and 2-amino-1-carbamoyl-3-oxo-3*H*-phenoxazine-8-carb­oxy­lic acid methyl ester (refcode MOQLUA; Graf *et al.*, 2007 ▸). Two sulfonamide derivatives were found, dimethyl 2,2′-[(3-oxo-3*H*-phenoxazine-1,9-di­yl)bis­(sulfonyl­imino)]di­acetate (refcode IGISOH; Bruyneel *et al.*, 2009 ▸) and 2-amino-*N*,*N*′-bis­(3-hy­droxy­prop­yl)-3-oxo-3*H*-phenoxazine-1,9-disulfonamide (ref­code IGISUN; Bruyneel *et al.*, 2009 ▸), but no sulfonates. The only metal complexes are two silver complexes (coordination through the phenoxazine N atom), *catena*-[(μ_3_-nitrato)(2-amino-3*H*-phenoxazin-3-one)silver(I)] (refcode BUVZOI; Pandurangan *et al.*, 2010 ▸) and bis­(2-amino-4a,7-dimethyl-4,4a-di­hydro-3*H*-phenoxazin-3-one)nitratosilver(I) (refcode ZEMYIC; Helios *et al.*, 2017 ▸). 2-Amino-3*H*-phenoxazin-3-one is also found as an inclusion with a hexa­nuclear iron(III) complex with no direct inter­actions between the cation and the phenoxazine mol­ecule (refcode CUMGEX; Feltham *et al.*, 2009 ▸).

A search of the Cambridge Structural Database (CSD, Version 5.42, update of November 2020; Groom *et al.*, 2016 ▸) for compounds with direct bonding between barium and an arene­sulfonate yielded 49 hits. Of these, none have three fused arene rings. Those with two fused rings and one sulfonate group include *catena*-(bis­{μ_2_-2-[2-(2-oxido-1-naphth­yl)diazenium­yl]naphthalene-1-sulfonato}­diaqua­barium dihydrate) (refcode CAWCUA; Kennedy *et al.*, 2012 ▸), *catena*-[(μ_4_-8-oxy-7-iodo­quinoline-5-sulfonato)­tri­aqua­barium monohydrate] (refcode IPUSUH; Mu­thiah *et al.*, 2003 ▸), *catena*-[(μ_3_-8-oxyquinoline-5-sulfonato)(μ_2_-aqua)­tri­aqua­barium] (ref­code NUVROM; Balasubramani *et al.*, 2010 ▸), and *catena*-[bis­(μ_3_-5,6-bi­hydroxy­flavone-6-sulfonato)­barium] (refcode PEHROK; Zhang *et al.*, 2006 ▸). Fused bicyclics with two sulfonate groups include [μ_2_-7-oxo-8-(phenyl­hydrazono)-7,8-di­hydro­naphthalene-1-sulfonate-3-sulfonato]­tetra­deca­aqua­dibarium dihydrate (refcode DEHMUZ; Kennedy *et al.*, 2006 ▸), *catena*-[(μ_8_-[2-(naphthalen-1-yl)hydrazinyl­idene]-7-oxo-7,8-di­hydro­naphthalene-1,3-di­sulfonato)­tris­(aqua)(*N*,*N*-di­methyl­formamide)­barium] (refcode EGOGUF; Black *et al.*, 2019 ▸), *catena*-[(μ_6_-1,5-naphthalene­disulfonato)­diaqua­barium] (refcode FOBYAX; Gao *et al.*, 2005*b* ▸), *catena*-[bis­(μ_7_-9,10-dioxoanthracene-2,6-di­sulfonato)­barium] (refcode IWIMUX; Platero-Prats *et al.*, 2011 ▸), bis­(6-ammonio­naphthalene-1,3-di­sulfonato)­hexa­aqua­barium tetra­hydrate (refcode NIHSIG; Gunderman *et al.*, 1997 ▸), *catena*-[(μ_6_-1,5-naphthalene­disulfonato)(μ_2_-di­aqua)­barium] (refcode RAKZEI; Cai *et al.*, 2001 ▸), and *catena*-[(μ_5_-naphthalene-2,7-di­sulfonato)(μ_2_-aqua)aqua­barium] (refcode YAFWEI; Huo *et al.*, 2004 ▸)

## 5. Synthesis and crystallization

A 2.05 g (9.89 mmol) sample of 3-amino-4-hy­droxy­benzene­sulfonic acid monohydrate (Aldrich, 98%) was dissolved in 100 ml of water. To this solution was added a cloudy suspension of 2.09 g (6.62 mmol) of Ba(OH)_2_^.^8H_2_O (Baker, >99%) in 50 ml of water. The resulting golden-brown cloudy mixture was stirred for about 30 minutes with gentle heating and then vacuum filtered. The resulting clear light orange–brown solution was transferred to a porcelain evaporating dish that was set out to evaporate in a fume hood. After several days, the water had completely evaporated leaving behind a polycrystalline brown crust. A few small (*ca.* 1 mm) clusters of red needle-shaped crystals were found and collected by hand. These were identified as (I) through the single crystal X-ray study. A powder X-ray diffraction pattern of the crust shows it to be partially crystalline with the most intense peak having a *d*-spacing of 15.48 Å. The 001 *d*-spacing for (I) is 15.79 Å, so this observation suggests another layered structure such as barium 3-amino-4-hy­droxy­benzene­sulfonate, but no single crystals of such a compound have been achieved to date. Subsequent attempts to repeat the synthesis of (I) by similar means were unsuccessful. Analysis of the starting 3-amino-4-hy­droxy­benzene­sulfonic acid by mass spectrometry yielded only a large peak at 189 amu due to 3-amino-4-hy­droxy­benzene­sulfonic acid and no evidence of 2-amino-3-oxo-3*H*-phenoxazine-8-sulfonic acid (292 amu). We have also attempted to perform the reaction hydro­thermally. So far, crystals of (I) have not been obtained. Mass spectral analysis of a solution obtained from such a reaction showed only the peak at 189 amu with none at 292. As a result, it remains unknown whether the tricyclic sulfonate formed *in situ* during the reaction or was already present in trace (*i.e.*, undetectable) amounts in the starting material. 2-Amino-3*H*-phenoxazin-3-one has been reported to form *in situ via* oxidative condensation of 2-amino­phenol (Feltham *et al.*, 2009 ▸).

## 6. Refinement

Crystal data, data collection and structure refinement details are summarized in Table 2 ▸. Hydrogen atoms bonded to carbon atoms were located in difference electron-density maps, constrained on idealized positions, and included in the refinement as riding atoms with C—H = 0.95 Å and their *U*_iso_ constrained to be 1.2 times the U_eq_ of the bonding atom. Oxygen- and nitro­gen-bound hydrogen atoms were located in difference electron-density maps and refined with distances restrained to O—H = 0.84 (1) Å and N—H = 0.88 (1) Å and *U*_iso_(H) = 1.5*U*_eq_(O or N).

## Supplementary Material

Crystal structure: contains datablock(s) I, global. DOI: 10.1107/S2056989025008242/jy2066sup1.cif

Structure factors: contains datablock(s) I. DOI: 10.1107/S2056989025008242/jy2066Isup2.hkl

CCDC reference: 2489654

Additional supporting information:  crystallographic information; 3D view; checkCIF report

## Figures and Tables

**Table 1:** Hydrogen-bond geometry (Å, °)

*D*—H⋯*A*	*D*—H	H⋯*A*	*D*⋯*A*	*D*—H⋯*A*
N21—H1*N*1⋯O4*W*^i^	0.87 (1)	2.50 (2)	3.089 (3)	125 (2)
N21—H2*N*1⋯O2*S*1^ii^	0.87 (1)	2.45 (2)	3.055 (2)	127 (2)
O1*W*—H1*A*⋯O1*S*1^iii^	0.84 (1)	1.96 (1)	2.787 (2)	168 (3)
O1*W*—H1*B*⋯O2*S*2^i^	0.84 (1)	1.95 (1)	2.757 (2)	163 (3)
O2*W*—H2*A*⋯O2*S*2^iv^	0.84 (1)	1.95 (1)	2.756 (2)	160 (3)
O2*W*—H2*B*⋯O1*S*2^v^	0.84 (1)	2.15 (2)	2.905 (2)	149 (3)
O3*W*—H3*A*⋯O31^vi^	0.83 (1)	2.11 (1)	2.906 (2)	162 (3)
O3*W*—H3*B*⋯O3*S*1^vii^	0.84 (1)	2.00 (1)	2.836 (2)	178 (3)
O4*W*—H4*A*⋯O2*S*1^vii^	0.84 (1)	2.04 (2)	2.823 (2)	155 (3)
O4*W*—H4*B*⋯O3*S*1^viii^	0.84 (1)	1.92 (1)	2.734 (2)	164 (3)
N22—H1*N*2⋯O2*W*^iii^	0.88 (1)	2.33 (2)	2.939 (2)	126 (2)

Symmetry codes: (i) 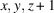; (ii) 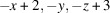; (iii) 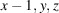; (iv) 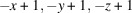; (v) 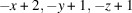; (vi) 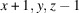; (vii) 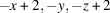; (viii) 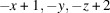.

**Table 2:** Experimental details

Crystal data	
Chemical formula	[Ba(C_12_H_7_N_2_O_5_S)_2_(H_2_O)_4_]
*M*_r_	791.91
Crystal system, space group	Triclinic, *P* 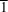
Temperature (K)	130
*a*, *b*, *c* (Å)	6.0052 (3), 13.9739 (6), 16.0374 (7)
α, β, γ (°)	79.966 (1), 87.604 (1), 85.581 (1)
*V* (Å^3^)	1320.72 (10)
*Z*	2
Radiation type	Mo *K*α
μ (mm^−1^)	1.75
Crystal size (mm)	0.21 × 0.11 × 0.04

Data collection	
Diffractometer	Bruker duo with Photon II area detector
Absorption correction	Multi-scan (*SADABS*; Krause *et al.*, 2015 ▸)
*T*_min_, *T*_max_	0.670, 0.746
No. of measured, independent and observed [*I* > 2σ(*I*)] reflections	49891, 8091, 7383
*R*_int_	0.048
(sin θ/λ)_max_ (Å^−1^)	0.715

Refinement	
*R*[*F*^2^ > 2σ(*F*^2^)], *wR*(*F*^2^), *S*	0.025, 0.056, 1.05
No. of reflections	8091
No. of parameters	442
No. of restraints	12
H-atom treatment	H atoms treated by a mixture of independent and constrained refinement
Δρ_max_, Δρ_min_ (e Å^−3^)	1.13, −0.67

Computer programs: *APEX3* and *SAINT* (Bruker, 2015 ▸), *SHELXT2014/5* (Sheldrick, 2015*a* ▸), *SHELXL2019/2* (Sheldrick, 2015*b* ▸) and *CrystalMaker* (Palmer, 2014 ▸).

## supplementary crystallographic information

### Bis(2-amino-3-oxo-3H-phenoxazine-8-sulfonato)tetraaquabarium Crystal data

**Table:**

[Ba(C_12_H_7_N_2_O_5_S)_2_(H_2_O)_4_]	*Z* = 2
*M**_r_* = 791.91	*F*(000) = 788
Triclinic, *P*1	*D*_x_ = 1.991 Mg m^−^^3^
*a* = 6.0052 (3) Å	Mo *K*α radiation, λ = 0.71073 Å
*b* = 13.9739 (6) Å	Cell parameters from 7993 reflections
*c* = 16.0374 (7) Å	θ = 2.6–28.3°
α = 79.966 (1)°	µ = 1.75 mm^−^^1^
β = 87.604 (1)°	*T* = 130 K
γ = 85.581 (1)°	Needle, red
*V* = 1320.72 (10) Å^3^	0.21 × 0.11 × 0.04 mm

### Bis(2-amino-3-oxo-3H-phenoxazine-8-sulfonato)tetraaquabarium Data collection

**Table:**

Bruker duo with Photon II area detector diffractometer	7383 reflections with *I* > 2σ(*I*)
Radiation source: fine-focus sealed tube	*R*_int_ = 0.048
phi and ω scans	θ_max_ = 30.5°, θ_min_ = 1.8°
Absorption correction: multi-scan (SADABS; Krause *et al.*, 2015)	*h* = −8→8
*T*_min_ = 0.670, *T*_max_ = 0.746	*k* = −19→19
49891 measured reflections	*l* = −22→22
8091 independent reflections	

### Bis(2-amino-3-oxo-3H-phenoxazine-8-sulfonato)tetraaquabarium Refinement

**Table:**

Refinement on *F*^2^	Primary atom site location: dual
Least-squares matrix: full	Secondary atom site location: difference Fourier map
*R*[*F*^2^ > 2σ(*F*^2^)] = 0.025	Hydrogen site location: mixed
*wR*(*F*^2^) = 0.056	H atoms treated by a mixture of independent and constrained refinement
*S* = 1.05	*w* = 1/[σ^2^(*F*_o_^2^) + (0.0203*P*)^2^ + 1.027*P*] where *P* = (*F*_o_^2^ + 2*F*_c_^2^)/3
8091 reflections	(Δ/σ)_max_ = 0.002
442 parameters	Δρ_max_ = 1.13 e Å^−^^3^
12 restraints	Δρ_min_ = −0.67 e Å^−^^3^

### Bis(2-amino-3-oxo-3H-phenoxazine-8-sulfonato)tetraaquabarium Special details

**Table:**

Geometry. All esds (except the esd in the dihedral angle between two l.s. planes) are estimated using the full covariance matrix. The cell esds are taken into account individually in the estimation of esds in distances, angles and torsion angles; correlations between esds in cell parameters are only used when they are defined by crystal symmetry. An approximate (isotropic) treatment of cell esds is used for estimating esds involving l.s. planes.

### Bis(2-amino-3-oxo-3H-phenoxazine-8-sulfonato)tetraaquabarium Fractional atomic coordinates and isotropic or equivalent isotropic displacement parameters (Å^2^)

**Table:**

	*x*	*y*	*z*	*U*_iso_*/*U*_eq_	
O51	0.3303 (2)	0.16808 (10)	1.38758 (8)	0.0164 (3)	
C11	0.6916 (3)	0.08226 (14)	1.57101 (12)	0.0175 (4)	
H11	0.836095	0.052057	1.584032	0.021*	
C21	0.5515 (3)	0.10563 (13)	1.63518 (12)	0.0181 (4)	
C31	0.3199 (3)	0.14981 (14)	1.61698 (12)	0.0179 (4)	
C41	0.2540 (3)	0.16875 (14)	1.53044 (12)	0.0177 (4)	
H41	0.108218	0.196792	1.516586	0.021*	
C61	0.3969 (3)	0.16690 (14)	1.24208 (12)	0.0168 (3)	
H61	0.251059	0.196706	1.231089	0.020*	
C4A1	0.3980 (3)	0.14693 (13)	1.46868 (11)	0.0151 (3)	
C71	0.5352 (3)	0.14400 (14)	1.17708 (12)	0.0174 (4)	
H71	0.484532	0.157255	1.120621	0.021*	
C5A1	0.4733 (3)	0.14589 (13)	1.32389 (12)	0.0149 (3)	
C81	0.7500 (3)	0.10127 (13)	1.19368 (12)	0.0152 (3)	
C91	0.8266 (3)	0.08138 (13)	1.27518 (12)	0.0163 (3)	
H91	0.973489	0.052561	1.285600	0.020*	
C9A1	0.6876 (3)	0.10366 (13)	1.34277 (11)	0.0145 (3)	
C101	0.6252 (3)	0.10227 (13)	1.48543 (12)	0.0149 (3)	
N21	0.6038 (3)	0.09329 (14)	1.71724 (11)	0.0239 (4)	
H1N1	0.505 (4)	0.113 (2)	1.7532 (14)	0.036*	
H2N1	0.741 (2)	0.075 (2)	1.7318 (18)	0.036*	
N101	0.7649 (3)	0.08222 (12)	1.42448 (10)	0.0157 (3)	
O1S1	0.9154 (2)	0.15587 (10)	1.04338 (9)	0.0191 (3)	
O2S1	1.1371 (2)	0.03763 (11)	1.14120 (9)	0.0222 (3)	
O3S1	0.8110 (3)	−0.01000 (11)	1.08025 (9)	0.0223 (3)	
O31	0.1938 (3)	0.16867 (11)	1.67576 (9)	0.0249 (3)	
O1W	0.2716 (3)	0.27323 (12)	1.00024 (10)	0.0251 (3)	
H1A	0.179 (4)	0.2309 (16)	1.0151 (18)	0.038*	
H1B	0.255 (5)	0.3095 (18)	1.0367 (14)	0.038*	
O2W	0.9735 (2)	0.43513 (12)	0.89003 (10)	0.0231 (3)	
H2A	0.913 (4)	0.4902 (11)	0.8951 (18)	0.035*	
H2B	1.097 (3)	0.433 (2)	0.9129 (16)	0.035*	
O3W	1.0267 (3)	0.19924 (11)	0.84245 (10)	0.0258 (3)	
H3A	1.069 (5)	0.204 (2)	0.7922 (8)	0.039*	
H3B	1.075 (5)	0.1428 (11)	0.8641 (17)	0.039*	
O4W	0.5649 (3)	0.11088 (12)	0.90674 (12)	0.0283 (3)	
H4A	0.675 (3)	0.0709 (17)	0.905 (2)	0.042*	
H4B	0.453 (3)	0.079 (2)	0.9212 (19)	0.042*	
S81	0.91727 (8)	0.06830 (3)	1.10817 (3)	0.01487 (8)	
Ba1	0.68488 (2)	0.28977 (2)	0.92392 (2)	0.01472 (3)	
O32	0.5473 (3)	0.33725 (11)	0.75905 (9)	0.0261 (3)	
N22	0.1310 (3)	0.40900 (14)	0.71889 (12)	0.0258 (4)	
H2N2	−0.006 (2)	0.434 (2)	0.7089 (19)	0.039*	
H1N2	0.176 (5)	0.397 (2)	0.7711 (9)	0.039*	
C102	0.3854 (3)	0.39552 (13)	0.50872 (11)	0.0143 (3)	
C12	0.2266 (3)	0.41550 (14)	0.57226 (12)	0.0171 (3)	
H12	0.082655	0.444023	0.556221	0.021*	
C22	0.2758 (3)	0.39472 (14)	0.65600 (12)	0.0176 (4)	
C32	0.5032 (4)	0.35245 (14)	0.68288 (12)	0.0193 (4)	
C42	0.6625 (3)	0.33122 (14)	0.61839 (12)	0.0177 (4)	
H42	0.807115	0.302586	0.633441	0.021*	
C4A2	0.6067 (3)	0.35204 (13)	0.53611 (11)	0.0142 (3)	
C5A2	0.7075 (3)	0.35155 (13)	0.39284 (11)	0.0136 (3)	
C62	0.8696 (3)	0.33028 (14)	0.33357 (12)	0.0154 (3)	
H62	1.012769	0.301945	0.350928	0.019*	
C72	0.8195 (3)	0.35103 (13)	0.24865 (11)	0.0146 (3)	
H72	0.928616	0.337478	0.206867	0.018*	
C82	0.6071 (3)	0.39209 (12)	0.22473 (11)	0.0121 (3)	
C92	0.4462 (3)	0.41247 (13)	0.28428 (11)	0.0129 (3)	
H92	0.302611	0.439961	0.266712	0.015*	
C9A2	0.4937 (3)	0.39287 (12)	0.37015 (11)	0.0127 (3)	
O52	0.7609 (2)	0.33062 (10)	0.47675 (8)	0.0154 (2)	
N102	0.3316 (3)	0.41509 (11)	0.42864 (10)	0.0145 (3)	
O1S2	0.5607 (2)	0.52893 (9)	0.09565 (8)	0.0154 (2)	
O2S2	0.3090 (2)	0.39942 (10)	0.11252 (8)	0.0164 (3)	
O3S2	0.6984 (2)	0.36779 (10)	0.07087 (8)	0.0169 (3)	
S82	0.54016 (7)	0.42413 (3)	0.11675 (3)	0.01149 (8)	

### Bis(2-amino-3-oxo-3H-phenoxazine-8-sulfonato)tetraaquabarium Atomic displacement parameters (Å^2^)

**Table:**

	*U*^11^	*U*^22^	*U*^33^	*U*^12^	*U*^13^	*U*^23^
O51	0.0168 (6)	0.0182 (6)	0.0130 (6)	0.0024 (5)	−0.0002 (5)	−0.0011 (5)
C11	0.0214 (9)	0.0167 (8)	0.0138 (8)	−0.0003 (7)	−0.0027 (7)	−0.0007 (7)
C21	0.0259 (9)	0.0124 (8)	0.0158 (8)	0.0003 (7)	−0.0037 (7)	−0.0022 (7)
C31	0.0242 (9)	0.0127 (8)	0.0165 (9)	0.0003 (7)	0.0012 (7)	−0.0024 (7)
C41	0.0194 (9)	0.0151 (8)	0.0176 (9)	0.0007 (7)	0.0005 (7)	−0.0012 (7)
C61	0.0165 (8)	0.0160 (8)	0.0166 (8)	0.0006 (7)	−0.0016 (7)	−0.0003 (7)
C4A1	0.0188 (8)	0.0120 (8)	0.0140 (8)	−0.0005 (6)	−0.0016 (7)	−0.0011 (6)
C71	0.0203 (9)	0.0166 (8)	0.0146 (8)	−0.0004 (7)	−0.0008 (7)	−0.0011 (7)
C5A1	0.0167 (8)	0.0129 (8)	0.0147 (8)	−0.0014 (6)	0.0019 (7)	−0.0013 (6)
C81	0.0185 (8)	0.0132 (8)	0.0140 (8)	−0.0009 (7)	0.0021 (7)	−0.0030 (6)
C91	0.0176 (8)	0.0134 (8)	0.0173 (9)	0.0011 (7)	−0.0005 (7)	−0.0019 (7)
C9A1	0.0174 (8)	0.0121 (8)	0.0136 (8)	−0.0011 (6)	−0.0005 (6)	−0.0007 (6)
C101	0.0175 (8)	0.0109 (8)	0.0157 (8)	−0.0006 (6)	−0.0014 (7)	−0.0010 (6)
N21	0.0321 (10)	0.0252 (9)	0.0140 (8)	0.0056 (8)	−0.0037 (7)	−0.0050 (7)
N101	0.0171 (7)	0.0158 (7)	0.0136 (7)	0.0009 (6)	−0.0011 (6)	−0.0014 (6)
O1S1	0.0239 (7)	0.0155 (6)	0.0164 (6)	−0.0005 (5)	0.0021 (5)	0.0009 (5)
O2S1	0.0197 (7)	0.0268 (7)	0.0197 (7)	0.0048 (6)	−0.0012 (5)	−0.0057 (6)
O3S1	0.0263 (7)	0.0184 (7)	0.0238 (7)	−0.0041 (6)	0.0005 (6)	−0.0077 (6)
O31	0.0316 (8)	0.0260 (8)	0.0161 (7)	0.0042 (6)	0.0041 (6)	−0.0047 (6)
O1W	0.0302 (8)	0.0252 (8)	0.0229 (8)	−0.0105 (6)	0.0029 (6)	−0.0096 (6)
O2W	0.0166 (7)	0.0255 (8)	0.0279 (8)	0.0009 (6)	0.0011 (6)	−0.0075 (6)
O3W	0.0371 (9)	0.0190 (7)	0.0187 (7)	0.0069 (6)	0.0078 (6)	−0.0015 (6)
O4W	0.0225 (8)	0.0208 (8)	0.0437 (10)	−0.0013 (6)	0.0011 (7)	−0.0117 (7)
S81	0.0181 (2)	0.01306 (19)	0.0133 (2)	0.00013 (16)	0.00076 (16)	−0.00253 (15)
Ba1	0.02297 (6)	0.01131 (5)	0.00926 (5)	0.00232 (4)	0.00072 (4)	−0.00182 (3)
O32	0.0432 (9)	0.0251 (8)	0.0099 (6)	0.0014 (7)	−0.0047 (6)	−0.0035 (6)
N22	0.0343 (10)	0.0275 (9)	0.0158 (8)	−0.0036 (8)	0.0084 (7)	−0.0060 (7)
C102	0.0189 (8)	0.0112 (8)	0.0129 (8)	−0.0025 (6)	0.0000 (6)	−0.0014 (6)
C12	0.0214 (9)	0.0153 (8)	0.0148 (8)	−0.0008 (7)	0.0012 (7)	−0.0032 (7)
C22	0.0260 (9)	0.0134 (8)	0.0138 (8)	−0.0031 (7)	0.0028 (7)	−0.0031 (7)
C32	0.0321 (10)	0.0135 (8)	0.0124 (8)	−0.0036 (7)	−0.0015 (7)	−0.0019 (7)
C42	0.0246 (9)	0.0152 (8)	0.0128 (8)	−0.0005 (7)	−0.0034 (7)	−0.0005 (7)
C4A2	0.0185 (8)	0.0115 (8)	0.0124 (8)	−0.0027 (6)	0.0007 (6)	−0.0007 (6)
C5A2	0.0185 (8)	0.0118 (8)	0.0099 (7)	−0.0009 (6)	−0.0021 (6)	−0.0002 (6)
C62	0.0144 (8)	0.0168 (8)	0.0142 (8)	0.0021 (6)	−0.0009 (6)	−0.0013 (7)
C72	0.0157 (8)	0.0153 (8)	0.0121 (8)	0.0010 (6)	0.0013 (6)	−0.0017 (6)
C82	0.0154 (8)	0.0105 (7)	0.0104 (7)	−0.0002 (6)	−0.0009 (6)	−0.0015 (6)
C92	0.0140 (8)	0.0132 (8)	0.0110 (8)	0.0009 (6)	−0.0009 (6)	−0.0012 (6)
C9A2	0.0158 (8)	0.0106 (7)	0.0117 (8)	−0.0009 (6)	0.0002 (6)	−0.0020 (6)
O52	0.0177 (6)	0.0186 (6)	0.0088 (6)	0.0018 (5)	−0.0017 (5)	−0.0008 (5)
N102	0.0167 (7)	0.0136 (7)	0.0130 (7)	−0.0005 (6)	0.0007 (6)	−0.0023 (6)
O1S2	0.0186 (6)	0.0123 (6)	0.0144 (6)	0.0001 (5)	−0.0009 (5)	−0.0004 (5)
O2S2	0.0158 (6)	0.0189 (6)	0.0149 (6)	−0.0024 (5)	−0.0024 (5)	−0.0028 (5)
O3S2	0.0222 (7)	0.0188 (6)	0.0098 (6)	0.0049 (5)	0.0004 (5)	−0.0058 (5)
S82	0.01439 (19)	0.01129 (18)	0.00859 (18)	0.00041 (15)	−0.00066 (14)	−0.00169 (14)

### Bis(2-amino-3-oxo-3H-phenoxazine-8-sulfonato)tetraaquabarium Geometric parameters (Å, º)

**Table:**

O51—C4A1	1.355 (2)	O3W—H3A	0.828 (10)
O51—C5A1	1.369 (2)	O3W—H3B	0.838 (10)
C11—C21	1.370 (3)	O4W—Ba1	2.7162 (16)
C11—C101	1.420 (3)	O4W—H4A	0.835 (10)
C11—H11	0.9500	O4W—H4B	0.838 (10)
C21—N21	1.344 (2)	Ba1—O32	2.7571 (14)
C21—C31	1.497 (3)	Ba1—O3S2^i^	2.7702 (13)
C31—O31	1.236 (2)	Ba1—O1S2^ii^	2.8067 (13)
C31—C41	1.433 (3)	O32—C32	1.239 (2)
C41—C4A1	1.347 (3)	N22—C22	1.335 (3)
C41—H41	0.9500	N22—H2N2	0.878 (10)
C61—C71	1.372 (3)	N22—H1N2	0.875 (10)
C61—C5A1	1.383 (3)	C102—N102	1.314 (2)
C61—H61	0.9500	C102—C12	1.415 (3)
C4A1—C101	1.471 (3)	C102—C4A2	1.469 (3)
C71—C81	1.395 (3)	C12—C22	1.363 (3)
C71—H71	0.9500	C12—H12	0.9500
C5A1—C9A1	1.396 (3)	C22—C32	1.495 (3)
C81—C91	1.379 (3)	C32—C42	1.433 (3)
C81—S81	1.7706 (19)	C42—C4A2	1.352 (2)
C91—C9A1	1.404 (3)	C42—H42	0.9500
C91—H91	0.9500	C4A2—O52	1.355 (2)
C9A1—N101	1.384 (2)	C5A2—O52	1.372 (2)
C101—N101	1.315 (2)	C5A2—C62	1.384 (2)
N21—H1N1	0.873 (10)	C5A2—C9A2	1.402 (2)
N21—H2N1	0.872 (10)	C62—C72	1.383 (2)
O1S1—S81	1.4605 (14)	C62—H62	0.9500
O1S1—Ba1	2.7678 (14)	C72—C82	1.398 (2)
O2S1—S81	1.4487 (15)	C72—H72	0.9500
O3S1—S81	1.4515 (15)	C82—C92	1.379 (2)
O1W—Ba1	2.7291 (16)	C82—S82	1.7667 (18)
O1W—H1A	0.840 (10)	C92—C9A2	1.394 (2)
O1W—H1B	0.836 (10)	C92—H92	0.9500
O2W—Ba1	2.7394 (16)	C9A2—N102	1.380 (2)
O2W—H2A	0.840 (10)	O1S2—S82	1.4580 (13)
O2W—H2B	0.839 (10)	O2S2—S82	1.4632 (14)
O3W—Ba1	2.7337 (15)	O3S2—S82	1.4464 (13)
			
C4A1—O51—C5A1	119.21 (15)	O2W—Ba1—O32	88.93 (5)
C21—C11—C101	121.32 (18)	O4W—Ba1—O1S1	73.21 (5)
C21—C11—H11	119.3	O1W—Ba1—O1S1	97.06 (5)
C101—C11—H11	119.3	O3W—Ba1—O1S1	71.77 (4)
N21—C21—C11	125.30 (19)	O2W—Ba1—O1S1	101.52 (4)
N21—C21—C31	114.17 (18)	O32—Ba1—O1S1	146.96 (4)
C11—C21—C31	120.52 (17)	O4W—Ba1—O3S2^i^	128.27 (5)
O31—C31—C41	122.58 (18)	O1W—Ba1—O3S2^i^	73.73 (4)
O31—C31—C21	119.80 (18)	O3W—Ba1—O3S2^i^	128.33 (5)
C41—C31—C21	117.62 (17)	O2W—Ba1—O3S2^i^	73.68 (4)
C4A1—C41—C31	120.26 (18)	O32—Ba1—O3S2^i^	141.30 (4)
C4A1—C41—H41	119.9	O1S1—Ba1—O3S2^i^	71.52 (4)
C31—C41—H41	119.9	O4W—Ba1—O1S2^ii^	130.98 (4)
C71—C61—C5A1	118.90 (17)	O1W—Ba1—O1S2^ii^	69.03 (4)
C71—C61—H61	120.6	O3W—Ba1—O1S2^ii^	138.33 (4)
C5A1—C61—H61	120.6	O2W—Ba1—O1S2^ii^	70.80 (4)
C41—C4A1—O51	118.24 (17)	O32—Ba1—O1S2^ii^	71.44 (4)
C41—C4A1—C101	122.92 (17)	O1S1—Ba1—O1S2^ii^	141.60 (4)
O51—C4A1—C101	118.84 (16)	O3S2^i^—Ba1—O1S2^ii^	70.24 (4)
C61—C71—C81	120.16 (18)	C32—O32—Ba1	173.60 (15)
C61—C71—H71	119.9	C22—N22—H2N2	122 (2)
C81—C71—H71	119.9	C22—N22—H1N2	119 (2)
O51—C5A1—C61	117.58 (16)	H2N2—N22—H1N2	119 (3)
O51—C5A1—C9A1	119.94 (16)	N102—C102—C12	119.91 (17)
C61—C5A1—C9A1	122.48 (17)	N102—C102—C4A2	122.50 (17)
C91—C81—C71	120.80 (17)	C12—C102—C4A2	117.59 (16)
C91—C81—S81	120.81 (14)	C22—C12—C102	121.45 (18)
C71—C81—S81	118.33 (14)	C22—C12—H12	119.3
C81—C91—C9A1	120.05 (17)	C102—C12—H12	119.3
C81—C91—H91	120.0	N22—C22—C12	124.2 (2)
C9A1—C91—H91	120.0	N22—C22—C32	115.40 (18)
N101—C9A1—C5A1	122.79 (17)	C12—C22—C32	120.40 (17)
N101—C9A1—C91	119.61 (17)	O32—C32—C42	122.58 (19)
C5A1—C9A1—C91	117.60 (17)	O32—C32—C22	119.49 (18)
N101—C101—C11	120.34 (17)	C42—C32—C22	117.93 (16)
N101—C101—C4A1	122.32 (17)	C4A2—C42—C32	119.84 (18)
C11—C101—C4A1	117.33 (17)	C4A2—C42—H42	120.1
C21—N21—H1N1	118.4 (19)	C32—C42—H42	120.1
C21—N21—H2N1	119.4 (19)	C42—C4A2—O52	118.35 (17)
H1N1—N21—H2N1	121 (3)	C42—C4A2—C102	122.75 (17)
C101—N101—C9A1	116.88 (16)	O52—C4A2—C102	118.89 (15)
S81—O1S1—Ba1	149.36 (8)	O52—C5A2—C62	117.87 (16)
Ba1—O1W—H1A	139 (2)	O52—C5A2—C9A2	119.60 (16)
Ba1—O1W—H1B	110 (2)	C62—C5A2—C9A2	122.53 (16)
H1A—O1W—H1B	104 (3)	C72—C62—C5A2	118.83 (17)
Ba1—O2W—H2A	113 (2)	C72—C62—H62	120.6
Ba1—O2W—H2B	124 (2)	C5A2—C62—H62	120.6
H2A—O2W—H2B	104 (3)	C62—C72—C82	119.51 (16)
Ba1—O3W—H3A	133 (2)	C62—C72—H72	120.2
Ba1—O3W—H3B	119 (2)	C82—C72—H72	120.2
H3A—O3W—H3B	104 (3)	C92—C82—C72	121.23 (16)
Ba1—O4W—H4A	113 (2)	C92—C82—S82	117.84 (13)
Ba1—O4W—H4B	135 (2)	C72—C82—S82	120.88 (13)
H4A—O4W—H4B	107 (3)	C82—C92—C9A2	120.21 (16)
O2S1—S81—O3S1	112.57 (9)	C82—C92—H92	119.9
O2S1—S81—O1S1	113.16 (9)	C9A2—C92—H92	119.9
O3S1—S81—O1S1	112.03 (9)	N102—C9A2—C92	119.32 (16)
O2S1—S81—C81	106.37 (9)	N102—C9A2—C5A2	122.99 (16)
O3S1—S81—C81	106.52 (9)	C92—C9A2—C5A2	117.69 (16)
O1S1—S81—C81	105.54 (8)	C4A2—O52—C5A2	119.19 (14)
O4W—Ba1—O1W	74.44 (5)	C102—N102—C9A2	116.82 (16)
O4W—Ba1—O3W	71.44 (5)	S82—O1S2—Ba1^ii^	143.33 (8)
O1W—Ba1—O3W	145.84 (5)	S82—O3S2—Ba1^iii^	134.57 (8)
O4W—Ba1—O2W	150.67 (5)	O3S2—S82—O1S2	113.65 (8)
O1W—Ba1—O2W	134.67 (5)	O3S2—S82—O2S2	113.53 (8)
O3W—Ba1—O2W	79.48 (5)	O1S2—S82—O2S2	111.77 (8)
O4W—Ba1—O32	82.35 (5)	O3S2—S82—C82	106.38 (8)
O1W—Ba1—O32	97.41 (5)	O1S2—S82—C82	105.30 (8)
O3W—Ba1—O32	79.65 (5)	O2S2—S82—C82	105.34 (8)
			
C101—C11—C21—N21	−176.93 (19)	C102—C12—C22—N22	−177.79 (19)
C101—C11—C21—C31	2.3 (3)	C102—C12—C22—C32	1.8 (3)
N21—C21—C31—O31	−2.2 (3)	N22—C22—C32—O32	−3.0 (3)
C11—C21—C31—O31	178.51 (19)	C12—C22—C32—O32	177.45 (18)
N21—C21—C31—C41	177.72 (18)	N22—C22—C32—C42	177.13 (18)
C11—C21—C31—C41	−1.6 (3)	C12—C22—C32—C42	−2.4 (3)
O31—C31—C41—C4A1	179.92 (19)	O32—C32—C42—C4A2	−178.08 (19)
C21—C31—C41—C4A1	0.0 (3)	C22—C32—C42—C4A2	1.8 (3)
C31—C41—C4A1—O51	−178.56 (16)	C32—C42—C4A2—O52	−179.69 (16)
C31—C41—C4A1—C101	0.8 (3)	C32—C42—C4A2—C102	−0.5 (3)
C5A1—O51—C4A1—C41	−179.53 (17)	N102—C102—C4A2—C42	−179.59 (18)
C5A1—O51—C4A1—C101	1.1 (2)	C12—C102—C4A2—C42	−0.2 (3)
C5A1—C61—C71—C81	−0.7 (3)	N102—C102—C4A2—O52	−0.4 (3)
C4A1—O51—C5A1—C61	178.84 (16)	C12—C102—C4A2—O52	178.92 (16)
C4A1—O51—C5A1—C9A1	−1.2 (3)	O52—C5A2—C62—C72	−179.83 (16)
C71—C61—C5A1—O51	−178.93 (17)	C9A2—C5A2—C62—C72	0.3 (3)
C71—C61—C5A1—C9A1	1.1 (3)	C5A2—C62—C72—C82	−0.5 (3)
C61—C71—C81—C91	0.1 (3)	C62—C72—C82—C92	0.1 (3)
C61—C71—C81—S81	177.28 (15)	C62—C72—C82—S82	177.45 (14)
C71—C81—C91—C9A1	0.3 (3)	C72—C82—C92—C9A2	0.4 (3)
S81—C81—C91—C9A1	−176.85 (14)	S82—C82—C92—C9A2	−177.02 (13)
O51—C5A1—C9A1—N101	0.2 (3)	C82—C92—C9A2—N102	179.08 (16)
C61—C5A1—C9A1—N101	−179.84 (18)	C82—C92—C9A2—C5A2	−0.5 (3)
O51—C5A1—C9A1—C91	179.30 (16)	O52—C5A2—C9A2—N102	0.7 (3)
C61—C5A1—C9A1—C91	−0.8 (3)	C62—C5A2—C9A2—N102	−179.44 (17)
C81—C91—C9A1—N101	179.15 (17)	O52—C5A2—C9A2—C92	−179.67 (16)
C81—C91—C9A1—C5A1	0.0 (3)	C62—C5A2—C9A2—C92	0.1 (3)
C21—C11—C101—N101	177.72 (18)	C42—C4A2—O52—C5A2	−179.77 (16)
C21—C11—C101—C4A1	−1.4 (3)	C102—C4A2—O52—C5A2	1.0 (2)
C41—C4A1—C101—N101	−179.31 (18)	C62—C5A2—O52—C4A2	178.98 (16)
O51—C4A1—C101—N101	0.0 (3)	C9A2—C5A2—O52—C4A2	−1.2 (2)
C41—C4A1—C101—C11	−0.2 (3)	C12—C102—N102—C9A2	−179.38 (16)
O51—C4A1—C101—C11	179.20 (16)	C4A2—C102—N102—C9A2	0.0 (3)
C11—C101—N101—C9A1	179.86 (17)	C92—C9A2—N102—C102	−179.70 (16)
C4A1—C101—N101—C9A1	−1.0 (3)	C5A2—C9A2—N102—C102	−0.1 (3)
C5A1—C9A1—N101—C101	0.9 (3)	Ba1^iii^—O3S2—S82—O1S2	−95.22 (12)
C91—C9A1—N101—C101	−178.17 (17)	Ba1^iii^—O3S2—S82—O2S2	34.00 (14)
Ba1—O1S1—S81—O2S1	174.88 (14)	Ba1^iii^—O3S2—S82—C82	149.39 (10)
Ba1—O1S1—S81—O3S1	46.32 (18)	Ba1^ii^—O1S2—S82—O3S2	145.61 (11)
Ba1—O1S1—S81—C81	−69.19 (17)	Ba1^ii^—O1S2—S82—O2S2	15.51 (15)
C91—C81—S81—O2S1	−9.72 (18)	Ba1^ii^—O1S2—S82—C82	−98.36 (13)
C71—C81—S81—O2S1	173.06 (15)	C92—C82—S82—O3S2	−161.27 (14)
C91—C81—S81—O3S1	110.57 (16)	C72—C82—S82—O3S2	21.27 (17)
C71—C81—S81—O3S1	−66.65 (17)	C92—C82—S82—O1S2	77.81 (15)
C91—C81—S81—O1S1	−130.19 (16)	C72—C82—S82—O1S2	−99.65 (16)
C71—C81—S81—O1S1	52.58 (17)	C92—C82—S82—O2S2	−40.47 (16)
N102—C102—C12—C22	178.97 (18)	C72—C82—S82—O2S2	142.07 (15)
C4A2—C102—C12—C22	−0.4 (3)		

Symmetry codes: (i) *x*, *y*, *z*+1; (ii) −*x*+1, −*y*+1, −*z*+1; (iii) *x*, *y*, *z*−1.

### Bis(2-amino-3-oxo-3H-phenoxazine-8-sulfonato)tetraaquabarium Hydrogen-bond geometry (Å, º)

**Table:**

*D*—H···*A*	*D*—H	H···*A*	*D*···*A*	*D*—H···*A*
N21—H1*N*1···O4*W*^i^	0.87 (1)	2.50 (2)	3.089 (3)	125 (2)
N21—H2*N*1···O2*S*1^iv^	0.87 (1)	2.45 (2)	3.055 (2)	127 (2)
O1*W*—H1*A*···O1*S*1^v^	0.84 (1)	1.96 (1)	2.787 (2)	168 (3)
O1*W*—H1*B*···O2*S*2^i^	0.84 (1)	1.95 (1)	2.757 (2)	163 (3)
O2*W*—H2*A*···O2*S*2^ii^	0.84 (1)	1.95 (1)	2.756 (2)	160 (3)
O2*W*—H2*B*···O1*S*2^vi^	0.84 (1)	2.15 (2)	2.905 (2)	149 (3)
O3*W*—H3*A*···O31^vii^	0.83 (1)	2.11 (1)	2.906 (2)	162 (3)
O3*W*—H3*B*···O3*S*1^viii^	0.84 (1)	2.00 (1)	2.836 (2)	178 (3)
O4*W*—H4*A*···O2*S*1^viii^	0.84 (1)	2.04 (2)	2.823 (2)	155 (3)
O4*W*—H4*B*···O3*S*1^ix^	0.84 (1)	1.92 (1)	2.734 (2)	164 (3)
N22—H1*N*2···O2*W*^v^	0.88 (1)	2.33 (2)	2.939 (2)	126 (2)

Symmetry codes: (i) *x*, *y*, *z*+1; (ii) −*x*+1, −*y*+1, −*z*+1; (iv) −*x*+2, −*y*, −*z*+3; (v) *x*−1, *y*, *z*; (vi) −*x*+2, −*y*+1, −*z*+1; (vii) *x*+1, *y*, *z*−1; (viii) −*x*+2, −*y*, −*z*+2; (ix) −*x*+1, −*y*, −*z*+2.
